# Unravelling physical and radiobiological effects of proton boron fusion reaction with anionic metallacarboranes ([*o*-COSAN]^-^) in breast cancer cells

**DOI:** 10.1186/s13550-025-01199-6

**Published:** 2025-02-21

**Authors:** Ana Belchior, Bianca C. Alves, Edgar Mendes, Francisco Megre, Luís C. Alves, Pedro Santos, Kai Nishimura, Hiroyuki Nakamura, Francesc Teixidor, Clara Viñas, Jorge Miguel Sampaio, Fernanda Marques, Teresa Pinheiro

**Affiliations:** 1 Centro de Ciências e Tecnologias Nucleares, Instituto Superior Técnico, Universidade de Lisboa, Estrada Nacional 10, Bobadela LRS, 2695-066 Portugal; 2 Departamento de Engenharia e Ciências Nucleares, Instituto Superior Técnico, Universidade de Lisboa, Estrada Nacional 10, Bobadela LRS, 2695-066 Portugal; 3 School of Life Science and Technology, Tokyo Institute of Technology, 4259, Nagatsuta-cho, Midori-ku, Yokohama, 226-8503 Japan; 4 Laboratory for Chemistry and Life Science, Institute of Innovative Research, Tokyo Institute of Technology, R1-13, 4259 Nagatsuta-cho, Midori-ku, Yokohama, 226-8503 Japan; 5 Institut de Ciència de Materials de Barcelona (C.S.I.C.) Campus U.A.B, Bellaterra, Barcelona, 08193 Spain; 6 Laboratório de Instrumentação e Física Experimental de Partículas, Av. Prof. Gama Pinto 2, Lisboa, 1649-003 Portugal; 7 Faculdade de Ciências da Universidade de Lisboa, Rua Ernesto de Vasconcelos, Edifício C8, Lisboa, 1749-016 Portugal; 8 iBB – Instituto de Bioengenharia e Biociências, Instituto Superior Técnico, Universidade de Lisboa, Av. Rovisco Pais 1, Lisboa, 1049-001 Portugal

**Keywords:** Proton Boron Fusion Reaction (PBFR), Monte Carlo (MC) dose assessment, Metallacarboranes (COSAN), Radiosensitizers, Radiobiological enhancement, Breast cancer

## Abstract

**Background:**

Protons, which are considered low-LET (Linear Energy Transfer) radiation, have an average RBE (relative biological effectiveness) of 1.1, with a range from 0.7 to 1.6. Thus, increasing biological effectiveness is of high interest in radiation oncology, and one way to enhance this is by using radiosensitizers. The present work investigates the effectiveness of the proton boron fusion reaction (PBFR) at the cellular level, using the sodium salt of metallacarborane [3,3’-Co(C2B9H11)2]^−^ (Na[o-COSAN]) as the boron source, aiming to explore the potential of this type of boron clusters as a radiosensitizer for proton therapy. Therefore, the main goal was to test the hypothesis that loading the cells with boron will favour the PBFR at energies close to the Bragg peak. This would enhance the radiation-induced biological effects through the production of alpha-particles.

**Results:**

MDA-MB-231 breast cancer cells were used. Nuclear microscopy assessed [o-COSAN] uptake and distribution in single cells, while biodistribution was studied in tumor-bearing Balb/cSlc-nu/nu mice (MDA-MB-231 xenograft), with boron accumulation in target organs and tumor measured by ICP-OES. The cells were irradiated with a proton beam tuned to reach the PBFR resonance energy of 675 keV at the cell layer. DNA damage was assessed with the g-H2AX assay and cell survival with the clonogenic assay. Beam parameters and dose calibration curves using radiochromic films validated Monte Carlo dosimetry simulations. As expected, we observed higher biological damage in irradiated cells and the presence of [o-COSAN]^−^ potentiated the damage. These results translate into a lower cellular viability, indicating that DNA damage imposed colonies smaller than their non-irradiated counterparts. This suggests that these damages either took longer time to be repaired or made the cells undergo less efficient survival mechanisms.

**Conclusions:**

The radiosensitizing effect of [o-COSAN]^−^ by strategic cellular ^11^B placement and proton irradiation intensifies the DNA damage, making the nucleus particularly susceptible and thus increasing the destructive capability of alpha-particles, generated in the nuclear fusion reaction, which may lead to increased cell mortality.

## Introduction

Cancer is a major public health problem and the second leading cause of death worldwide [1]. Over the years, remarkable progress has been made to understand the complexity of cancer and the hallmarks of cancer development [2]. However, with its growing incidence, the management of cancer continues to be a challenge in the 21st century. Therapeutic application of ionizing radiation for treating tumors has a long history since the discovery of X-rays and the radioactivity [3]. Different types of ionizing radiation such as photons, neutrons, protons, and carbon ions have been used to treat malignant tumors. Radiation therapy (RT) is one of the main methods of treating tumors along with chemotherapy and remains an important treatment modality with ca. 50% of all patients receiving RT during their course of disease [4]. The main goal of RT is to use high doses of ionizing radiation to kill cancer cells. The mechanisms of radiation-induced cell death are multifactorial depending on the type of cancer cells, radiation dose and quality, time of radiation exposure, and tumor microenvironment. Radiation acts on cells, directly damaging DNA and inducing DNA double-strand breaks (DSBs), or indirectly generating reactive oxygen species (ROS) through the ionization of cellular water. The most common RT lethal effects include apoptosis and/or necrosis [3, 5, 6].

Although great success has been attained with RT, there are still the challenges to enhance radiation damage to tumors while reducing the side effects to normal cells and tissues. Radiosensitizers, usually small molecules or drugs can enhance the killing effect on tumor cells when combined with radiation, by increasing DNA damage and ROS production. In most cases, radiosensitizers are highly effective and have low toxicity and some of them have currently entered clinical studies [7, 8].

Proton therapy (PT) is a type of radiation therapy that uses protons rather than high-energy X-rays or gamma-rays. In principle, PT induces fewer side effects and less damage to other healthy tissues due to its ballistic nature, i.e., protons can target more directly the tumor site and can achieve a better dose distribution compared to photon-based RT. In fact, as charged particles penetrate into tissues they gradually lose energy along their path, with the majority of energy being deposited near the end of their range. This results in a dose distribution characterized by the Bragg peak, which is influenced by the proton’s energy and can vary in range. Thus, proton therapy is ideal when organ preservation is a priority [9]. However, several challenges and limitations persist that constrains the routine application of this treatment such as the expensive operation of proton facilities, their complexity and the RBE variations and uncertainties [10–13].

Proton boron fusion therapy (PBFT) involves the selective accumulation of a boron containing radiosensitizer in the tumor followed by proton irradiation. This approach aims to significantly increase the biological effectiveness of proton therapy. To this end, the PBFR, based on the proton fusion on the ^11^B stable isotope (representing 80% of natural boron) with the formation of three α-particles (one α-particle of 3.76 MeV and two α-particles of 2.74 MeV) together with a prompt gamma photon at 719 keV, and a high energy release (Q-value = 8.7 MeV), has been studied. In theory, this reaction which has a maximum of the cross-section at a proton energy of 675 keV, close to the Bragg peak, could significantly increase the absorbed dose in the target tumor volume. The main effect would be irreparable, highly localised DNA damage due to the generation of short range, densely ionising α-particles with a high linear energy transfer (LET). This is due to the increased clustering of lesions along the trajectories of the alpha particles [14–16]. These properties make PBFT particularly attractive, as the presence of ^11^B could concentrate the dose within the tumor, acting as an effective radiosensitizer for proton therapy.

The first experimental proof of PBFR has been demonstrated with the use of ^11^B carriers sodium borocaptate (BSH) and p-boronphenylalanine BPA, two boron compounds in clinical studies [17–20]. In addition, Ricciardi et al. [21] demonstrated that irradiation of human cancer cells in the presence of ^11^B compounds, at the proton-^11^B nuclear reaction resonance energy of 675 keV, resulted in the highest reduction in survival fraction reported to date. This supports the underlying basis of PBFT.

Despite the interesting experimental results, further investigation into the mechanisms of boron as radiosensitizers for proton therapy is needed [22]. More recently, a preliminary study on the biological effects generated by a PBFR with ferrabis(dicarbollide) (Na[*o*-FESAN]) in the U87 glioblastoma cells was reported [23]. This proof-of-concept study provides the first evidence that proton therapy efficacy is significantly enhanced in U87 cells when combined with boron-rich metallacarboranes. The efficient uptake of Na[o-FESAN] by U87 cells, followed by proton irradiation at 675 keV with a dose of 9 Gy, resulted in a significant loss of cell viability and survival. These results suggest that the combination of boron-rich compounds as radiosensitizers with PBFR may be a promising approach for the treatment of resistant tumours.

**Fig. 1:**
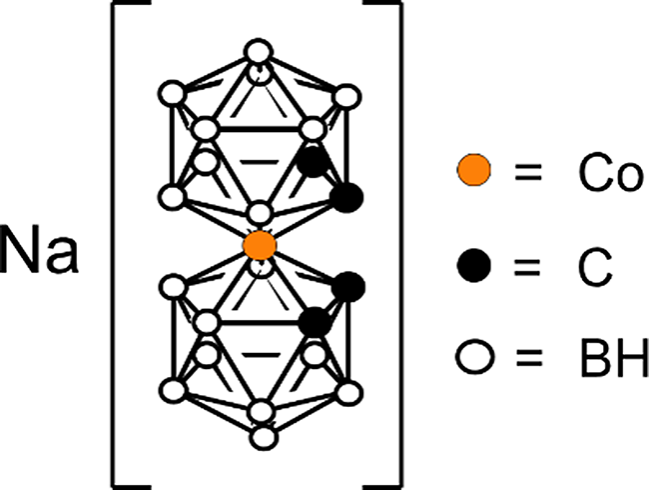
Molecular structure of the sodium salt of cobaltabisdicarbollide, Na[*o-*COSAN], herein ([*o*-COSAN]^-^) [24]

With the present study, we aim to contribute to the understanding of the extent of cellular damage and escalation of the effects induced by direct or secondary radiation upon PBFR using the sodium salt of cobaltabisdicarbollide, Na[*o-*COSAN], herein ([*o*-COSAN]^-^) [24] as the boron source (Fig. 1) on a breast cancer cell model.

The exploration of research accelerators capable of producing proton beams of a few MeV that can be focused to micrometre dimensions allows cell irradiation and imaging. These capabilities play an important role in studying the cellular uptake of new high boron compounds for proton therapy and the irradiation effects at energies in the Bragg peak region to evaluate cellular synergistic cell killing effects. Benchmarking of proton irradiation with low energy proton beams on cancer cell lines is of utmost interest for dosimetric studies to estimate the absorbed dose at the nanoscale and provide insights into the application of PBFR in cancer radiotherapy.

To make this technique a reality in clinical practice, it is necessary to establish the relationship between dosimetric physical quantities and the expected biological effects. Only with this knowledge can reliable treatment plans be achieved. Therefore, in this work, we dedicate special attention to the dosimetric characterization of the proton beam using beam parameters and radiochromic films. This characterization was used to validate our Monte Carlo (MC) simulations of the beamline, enabling us to determine the radiation field characteristics (e.g., dose distribution and energy spectra) at the point of cell irradiation.

Dosimetry is a major concern in this field, since it is used for quality equipment control and treatment planning. This can be done via direct or indirect measures. In the direct measures, ionisation chambers (IC) and radiochromic films (RCF) are at the upfront, while the indirect measures are done mostly by MC methods.

There are sources of uncertainty related to the spatial and temporal non-uniformity of the dose distribution. Therefore, computational methods based on MC simulations are needed for accurate dosimetry assessment. This will improve the assessment of subcellular dose distribution in cell models to validate cellular responses to irradiation, such as viability, survival (clonogenic) assays and DNA damage, which are the main endpoints of radiobiological effects.

The main objectives of this work are: (1) to evaluate the efficacy of [*o*-COSAN]^-^ cellular uptake in MDA-MB-231 breast cancer cells and its distribution and accumulation in target organs of MDA-MB-231 tumor-bearing mice; (2) to disentangle deleterious mechanisms in MDA-MB-231 cells by correlating genetic and cellular damage in [*o*-COSAN]^-^treated and proton-irradiated cells to shed light on the efficacy of [*o*-COSAN]^-^ in enhancing the Dose Modifying Factor (DMF) for protons; (3) MC simulations to improve dosimetry assessment. The combination of these three approaches can place [*o*-COSAN]^-^ in a relevant position as a new tool for enhancing biological damage caused by proton radiation.

## Methods

### Cell line and viability

MDA-MB-231 breast cancer cells were purchased from American Type Culture Collection (ATCC, HTB-26). Cells were cultured in DMEM medium supplemented with 10% fetal bovine serum (FBS) and incubated at 37ºC in a humidified 5% CO_2_ atmosphere. Na[*o*-COSAN] stock solutions at 1 mM were prepared in DMEM medium without supplements and kept at -20ºC. Cell viability was assessed using the MTT assay following a method similar to the one previously described [24]. Briefly, MDA-MB-231 cells (0.8 − 5 × 10^4^ cells/well) were seeded in 96-well plates and left to adhere. After 24 h, cells were incubated with increasing concentrations of Na[*o*-COSAN] from 1 µM to 1000 µM for 6 and 24 h. After incubation, the medium was removed, and each well was incubated with an MTT solution (0.5 mg/mL in medium) for 3 h at 37 °C. Then, the medium was discarded, and the formazan crystals were solubilized in 200 µL DMSO. Absorbance was measured at 570 nm using an Varioskan TM LUX microplate reader (Thermo Scientific, Waltham, MA, USA). The dose-response curve fit of the absorbance as a function of log10[concentration] was performed using GraphPad Prism software (version 9.5.1) to assess viability and IC50 (concentration at which half the cell population is expected to die) at 6 and 24 h.

## Cellular uptake by nuclear microscopy

MDA-MB-231 cells (10^4^-10^5^ cells) were seeded on 100 nm thick silicon nitride (Si_3_N_4_) membranes (Silson Ltd., UK), allowed to adhere, and incubated for 24 h in medium containing Na[*o*-COSAN] at 50 µM. The culture medium was removed and the monolayer of cells on the membranes was washed with cold PBS, immediately frozen at -80 °C, transferred to a cryostat at -25 °C and allowed to dry overnight for subsequent analysis of [*o*-COSAN]^−^ uptake [25]. The nuclear microscopy facility at the Van de Graaff accelerator of the Campus Tecnológico e Nuclear/Instituto Superior Técnico CTN/IST [26] was used to image and quantify the distribution of Co (as a signature of [*o*-COSAN]^−^) in single cells. Briefly, a 2.0 MeV focused proton beam of 3 × 4 µm^2^ was raster scanned across the target cell on the silicon nitride window, simultaneously delivering images of density by STIM (Scanning Transmission Ion Microscopy), elemental distributions by PIXE (Particle Induced X-ray Emission) and matrix constituents by EBS (Elastic Backscattering Spectroscopy). Spectra recorded and/or extracted from maps in selected regions of the cell allowed Co concentrations to be determined in major cell compartments, such as the cytoplasm and nucleus [25–27]. At least three cells treated with Na[*o*-COSAN] at a concentration of 50 µM were analyzed, and Co concentrations were measured using a minimum of five spectra acquired from both the nucleus and cytoplasm regions. The average concentrations for each of these regions were calculated based on the individual spectra. Data acquisition, spectra processing and concentration calculation were performed using the OMDAQ-3 program (Oxford Microbeams Ltd., UK) (http://www.microbeams.co.uk/ Accessed 12-6-2024). Elemental concentrations are expressed as µg/g dry weight.

## Biodistribution of [o-COSAN]− in the MDA-MB-231 xenograft model

MDA-MB-231 tumor-bearing mice (Balb/cSlc-nu/nu, female, 5–6 weeks old, 14–20 g, Sankyo Labo Service Co., Ltd., Japan) were prepared by injecting subcutaneously (s.c.) a suspension of MDA-MB-231 cells (8.0 × 10^5^ cells/mouse) directly into the right thigh. The mice were kept on a regular chow diet and water and maintained under 12 h light/dark cycle in an ambient atmosphere. When the tumor diameter became 5 to 7 mm, the mice were injected via the tail vein with a 200 µL PBS solution of Na[*o*-COSAN] (7.5 mgB/kg). At 1 h after injection, the mice were lightly anesthetized, and blood samples were collected by cardiac puncture. The mice were then sacrificed by cervical dislocation and dissected. Heart, liver, lung, kidney, spleen, muscle, brain and tumor were excised, washed with 0.9% NaCl solution, and weighed. The excised organs were digested with 1 mL of HNO_3_ at 90 °C for 1 h, and then the digested samples were diluted with distilled water. After filtering through a hydrophobic filter, boron concentration was measured by an inductively coupled plasma optical emission spectroscopy (ICP-OES, iCAP 7400 Duo, Thermo Fisher Scientific Inc., USA). All animal experiments were conducted in accordance with the Guide for the Care and Use of Laboratory Animals approved by the Animal Use Review Board and Ethical Committee of Tokyo Institute of Technology (Permission number: D2021007).

## Dosimetric studies

### Calibration curve of XR-RV3 radio-chromic films, using 60Co gamma-rays

Radiochromic films, such as GafChromic^R^ XR-RV3 [28], are extensively used for passive dosimetry due to their near tissue equivalence, high spatial resolution, and ease of handling [29].

A Dose calibration curve, for XR-RV3 films (Ashland, USA; dose range 0.01 Gy to 30 Gy) both in their native laminated form and unlaminated (with the outer yellow polyester and adhesive layers removed), was done in a calibrated position of the experimntal ^60^Co irradiator in operation at CTN/IST (Campus Tecnológico e Nuclear/Instituto Superior Técnico [30] at doses of 1, 2, 6, and 10 Gy. Two discs were irradiated at each dose and an unexposed disc was used as a reference for background measurements. After irradiation all discs, including the references, were stored in a light-tight box at room temperature, for 48 h, to stabilize their response. For measuring the radiation induced optical density alterations of the films, an Epson Perfection V850 Pro scanner was used in transmission mode. To ensure consistency, each disc was placed in the same position on the scanner window, with the yellow polyester or active layer side down between the scanner glass and a microscope slide. Each disc was scanned six times.

The scanned images were processed using ImageJ software. Fixed size regions of interest (ROI) were defined on each image, and the mean pixel values and standard deviations were calculated for the red, green, and blue (RGB) channels. These values were converted to net optical density (netOD) relative to the unexposed reference film [31]. The *net*OD values as a function of dose were fitted, according to Sanchez-Parcerisa et al. [32], using the equation:

1 $$\:netOD\left(Dose\right)=A\times\:Dose+B\times\:{Dose}^{C}$$

where A (Gy^-1^), B (Gy^-C^), and C are fitting parameters. The parameters were estimated using the Python implementation of the Levenberg-Marquardt algorithm.

### Proton dose calculation

For proton experiments, the outer layers of the XR-RV3 film were removed due to the mismatch between proton range and layer thickness of ~ 110 μm [33]. The measurement of the *net*OD values, of XR-RV3, was done as outlined in the previous section. The dose was, then, calculated using Eq. 1 and parameters A, B and C from the Co-60 calibration curve.

## MC dose calculation

MC simulations were performed using TOPAS (TOol for PArticle Simulation), a toolkit based on GEANT4 code [34]. The aim of these simulations were (i) validation of the irradiation setup based on dosimetric calculations, (ii) Proton dose calculations, and (iii) check whether the resonance energy of 675 keV (corresponding to the maximum value of the proton-^11^B nuclear reaction cross section) is reached in the irradiated cell layer.

The proton beam irradiation setup at the external microbeam facility of the CTN/IST Van de Graaff accelerator consists of an exit nozzle (2.7 mm internal diameter) with a 6.3 μm thick Mylar window to extract the beam from the vacuum chamber into the air and a x-y-z stage, which allows samples to be positioned at a defined distance from the exit nozzle oriented perpendicular to the proton beam path. Using the imaging capabilities of the nuclear microprobe, a microscopy grid positioned close to the exit nozzle is used for fine tuning of the beam focusing (tens of micrometer size) and for limiting the scanned area to the exit nozzle dimensions. In this way, the beam current crossing the nozzle exit foil matches the beam current measured inside the vacuum chamber [35].

**Fig. 2:**
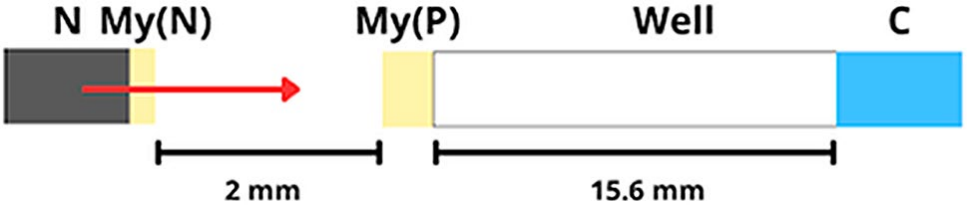
Geometry of the beamline implemented in the MC simulation considering the four main components: N, Nozzle (Cylinder in vacuum, radius = 1.35 mm and length = 2 nm); My(N), Mylar Nozzle (Cylinder in Mylar: radius = 1.75 mm and length = 6.3 μm); My(P), Mylar Plate (Cylinder in Mylar: radius = 2.5 mm and length = 12.6 μm) and C, Cells (water cylinder: radius = 2.15 mm and length of 30 μm)

The geometry of the proton beamline was implemented in the simulation considering the four main components shown in Fig. 2. These elements are the nozzle to extract the beam to air, the Mylar foil at the nozzle exit, the Mylar foil at the entrance of the cell culture plate, and a column of water at the bottom of the plate well (corresponding to the irradiated cell culture). The four elements were created in a world of air. The nozzle was made as a cylinder of vacuum with the same diameter as the one on the accelerator. The diameter of both monolayers of Mylar were some millimetres larger than the nozzle and the well, respectively, to ensure that the beam crossed both layers before reaching the cell medium. This medium was designed as a volume with the diameter of the well and 30 μm thickness of water.

A 2 MeV proton beam with 10% spread was simulated according to the geometry of the irradiation setup. The angular spread was defined to match the area of the plate well. The number of particles used for simulation were in order of 10^6^, of which, approximately, 97% reach the cells (C) layer (see Fig. 2). As shown by Qi Tan et al. [36], the module of electromagnetic physics *StandardPhysics_option4* seems to be accurate and fast for simulating trajectories of low energy protons (< 4 MeV).

To simulate the response of the unlamnitated and laminated XR-RV3 films we implemented the composition of each layer based on data from Setilo et al. [28]. The water cylinder in Fig. 2 was replaced by the layered geometry of the films, where the active layer was placed at the position corresponding to the water cylinder entrance.

In this case, the unlaminated films were irradiated in the same spot were the bottom of the well would be, in the cell irradiation. This values would later be converted in absorved dose in water, using the calibration curve obtained with the films, according to Eq. 1.

Due to the stopping of the low energy protons in the polyester and adhesive layers, only the unlaminated films were used in the experimental irradiation. However, the irradiation with laminated and unlaminated films were made in MC TOPAS (TOol for PArticle Simulation). This would give the order of magnitude for the attenuation in both polyester and adhesive layer.

## Radiobiological studies

### Cell irradiation with protons

Proton irradiation was performed in air using the external microbeam facility at the Van de Graaff accelerator of the CTN/IST. Briefly, a focused 2.0 MeV proton beam of ∼70 × 70 µm^2^ was used and rastered scanned over a cell monolayer placed in a cell plate, positioned prependicular to the proton beam path. The raster scan duration and the irradiation time of 10 s ensured irradiation homogeneity [35]. The maximum achievable scan area is in the range of 0.08–0.13 cm^2^ (corresponding to 40% of the total area of one well, 0.32 cm^2^, in a 96-well plate). A proton beam current in the pA range was used, corresponding to a proton intensity of 2–6 × 10^6^ protons/s. Prior to irradiation, part of the culture medium was removed, as described elsewhere [33]. The proton beam energy was tuned to 675 keV (main resonance energy of the proton-boron nuclear reaction) within the cell layer by controlling the air path length and using Mylar^R^ foils as attenuators. The details of the irradiation geometry are described above and illustrated in Fig. 2.

### Cellular survival

MDA-MB-231 cells were seeded in each well of a 6 well plate and incubated for 24 h. Cells were incubated with 50 and 100 µM of Na[*o-*COSAN], for 24 h at 37 °C, and then irradiated with protons, as described in 2.3.1. After irradiation, fresh medium was added and cells were incubated for 7 days in the same conditions. After 7 days of incubation, the medium was aspirated and cells were fixed and stained with Giemsa’s azur eosin methylene blue solution diluted 5% v/v in buffer solution. Finally, colonies were manually counted. Moreover, a CellProfiler pipeline was used to check the size of the different colonies observed in order to assess whether proton irradiation interfered with the capacity of replication of MDA-MB-231 cells.

### Foci quantification

MDA-MB-231 cells were seeded at a density of 10,000 cells per well in an eight-well chamber slide and allowed to attach overnight. Cells were incubated with 50 and 100 µM of Na[*o-*COSAN], for 24 h at 37 °C, and then irradiated with protons, as described in 2.4. MDA-MB-231 cells were washed three times with PBS and fixed with 4% formaldehyde in PBS for 15 min. After washing with PBS, cells were permeabilized with Triton X-100 (0.5%) at room temperature for 5 min followed by two washing steps with 1% BSA in PBS. Then, cells were incubated with an anti-γ-H2AX primary antibody (mouse anti-γ-H2AX (ser139), Stressgen) at 2 µg/mL for 1 h. After being washed twice with 1% BSA in PBS, cells were incubated with a Texas Red-X-conjugated antimouse secondary antibody at 1 mg/mL for 1 h, followed by three washing steps with PBS. Cells were finally mounted in anti-fade mounting media with DAPI (Vectashield H-1200, Vector Laboratories). Cells were analyzed at under ×64 magnification. Several high-quality 2D images were randomly collected in each slide and analyzed using a modified Speckle Count pipeline from the freeware CellProfiler [37]. At least 200 nuclei were analyzed per experiment per dose. Statistical analysis was performed with the GraphPAD Prism software.

## Results

### Imaging and quantifying [o-COSAN]− uptake in single cells

**Fig. 3:**
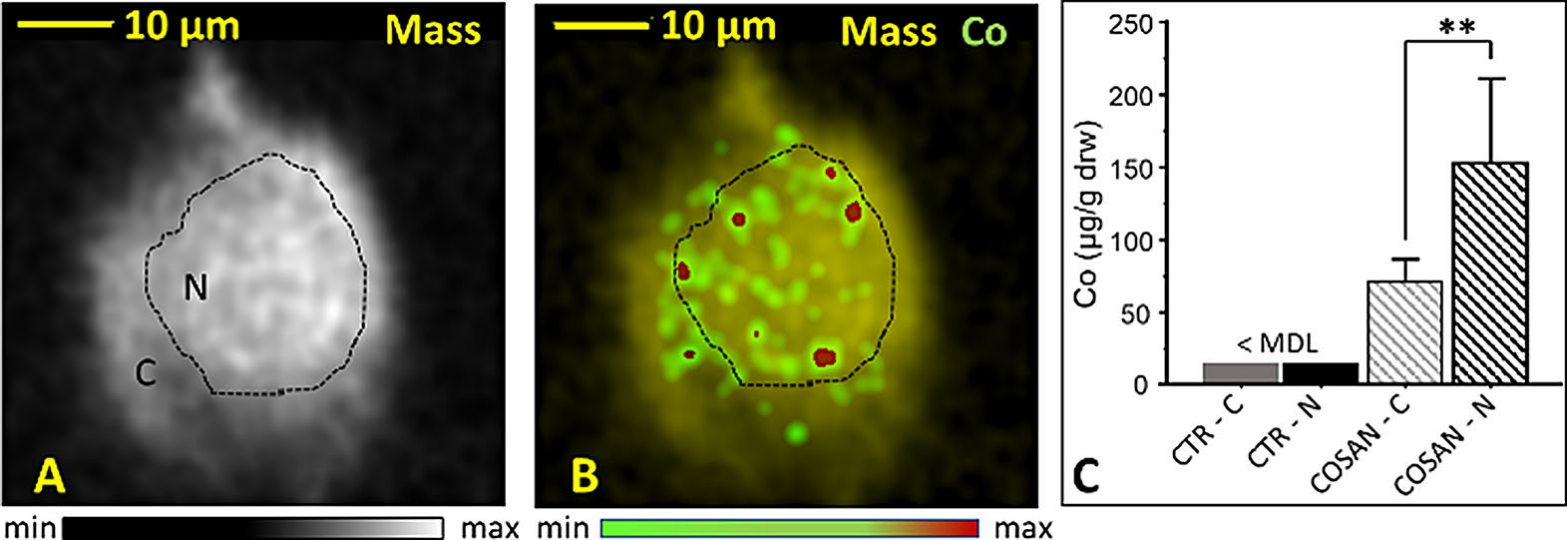
Cobalt (Co) as a signature of [*o*-COSAN]^*−*^ uptake in MDA-MB-231 single cells. Nuclear microscopy images of mass density and Co distribution in MDA-MB-231 cells incubated for 24 h with 50 µM Na[*o*-COSAN]: (**A**) Mass density (Mass); (**B**) Mass density overlapping with Co, showing a preferential accumulation of Co in the nuclear region; (**C**) Quantitative Co concentration in the cytoplasm and nucleus of controls (CTR) and Na[*o*-COSAN] treated cells (for each condition, average of data extracted from *N* ≥ 3 single cells); Minimum detection limit (MDL) for Co ~ 20 µg/g dry weight (dw). Cytoplasm (C) and nucleus (N). The dotted line in A and B, delineates the high density nuclear region

The uptake of [*o*-COSAN]^−^ in single MDA-MB-231 cells was analysed using a combination of nuclear microscopy techniques: STIM, PIXE, and EBS. These techniques allow correlative imaging and quantification of elemental distributions. STIM produced mass density images that revealed cell morphology, including high-density nuclei and low-density cytoplasm. These morphological details were essential for correlating with the elemental distributions obtained from PIXE and assessing localization. Figure 3, shows that the Co distribution map, which serve as a signature of [*o*-COSAN]^−^ uptake, displayed significant accumulation in the cells after 24 h of incubation with 50 µM Na[*o*-COSAN]. Co showed higher accumulation towards the nuclear region, as confirmed by significantly higher concentrations of Co in the nucleus compared to the cytoplasm (Fig. 3C). In contrast, untreated cells did not show detectable Co levels, which are below the PIXE detection limit (~ 20 µg/g dry weight). Therefore, considering the stoichiometry of the carborane cluster [C_2_B_9_H_11_]^−2^.in the [*o*-COSAN]^−^ molecule [24], the boron-11 (^11^B) content can be estimated. For example, at an average Co concentration of 153 µg/g in the nucleus (Fig. 3C), the ^11^B content is 425 µg/g, corresponding to approximately 2.3 × 10^9 11^B atoms in the nuclear volume of a single cell. This is important information provided by nuclear microscopy. It will be needed for future advanced dosimetry studies, including PBFR.

[*o*-COSAN]^−^ is remarkably stable, water-soluble, and interact with various biological molecules, including DNA [38–40]. The nuclear microscopy techniques—STIM, PIXE, and EBS— results are consistent with the ones obtained using Synchrotron Radiation-Fourier Transform Infrared (FTIR) micro-spectroscopy (SR-FTIRM) at the MIRAS Beamline of the ALBA synchrotron light source. These SR-FTIRM techniques were applied to study two different glioma-initiating cell (GIC) types: mesenchymal and proneural, following their uptake of Na[o-COSAN]. Analysis of the SR-FTIRM spectroscopic data, particularly in the DNA spectral regions, suggests that Na[*o*-COSAN] strongly interacts with DNA strands after cellular uptake. Furthermore, the mapping data indicate the nuclear localization of [*o*-COSAN]⁻ [38]. To shed light on the mechanism underlying the observed translocation of [*o*-COSAN]⁻ anions across membranes, molecular dynamics simulations were performed [41]. The simulations revealed that the permeation of [*o*-COSAN]⁻ anions through a lipid bilayer occurs in a cooperative manner. [*o*-COSAN]⁻ anions can efficiently traverse bilayers that are otherwise impermeable to ions through a cooperative flip-flop mechanism. This process is facilitated by the formation of transient, elongated structures within the membrane, suggesting a novel ion permeation pathway with self-assembling properties.

### Cellular viability and IC50 calculation

**Fig. 4:**
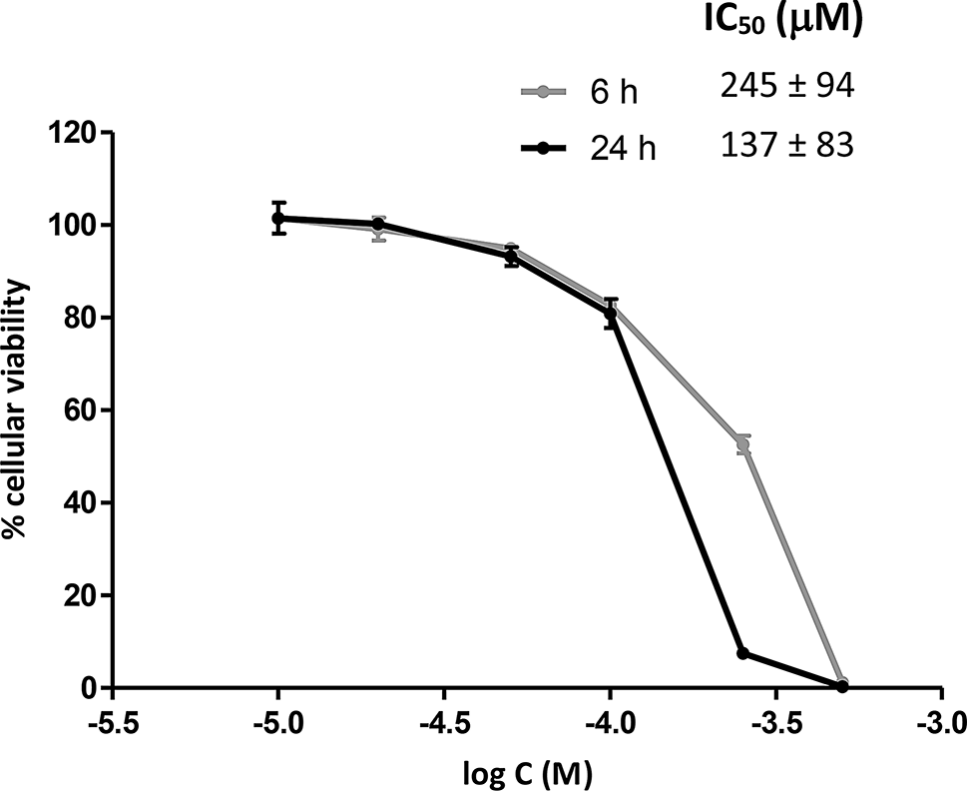
Dose-response curves obtained from the GraphPad Prism software to calculate the IC_50_ values of Na[*o*-COSAN] at 6 h and 24 h incubation. Results are mean ± SD of two independent experiments

Prior to the irradiation studies, we screened the cytotoxic activity of the Na[*o*-COSAN] in MDA-MB-231 cells, using the MTT assay to assess the viability of the cells treated with increasing concentrations, in the range of 1-1000 µM. This study was expected to guide the selection of adequate concentrations of the Na[*o*-COSAN], as the [*o*-COSAN]- should not exhibit intrinsic cytotoxic activity at the concentrations used for the evaluation of their radiosensitizing capabilities. Figure 4 shows the IC50 values determined from dose-response curves after 6 h and 24 h incubation with Na[*o*-COSAN].

### Biodistribution

**Fig. 5:**
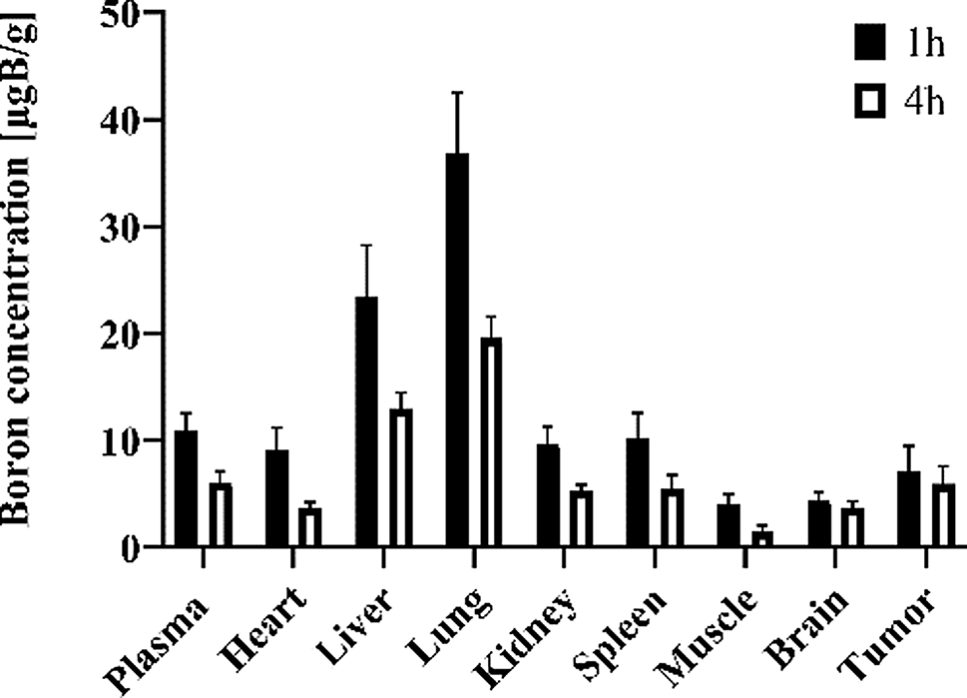
Biodistribution studies of Na[*o*-COSAN] (7.5 mg B/kg) in MBA-MB-231 xenograft model mice Balb/cSlc-nu/nu, female (mean ± SD, *n* = 6 mice), after 1 and 4 h post-injection

The biodistribution studies provide important preclinical information regarding the identity of the potential target organs and tissues. Na[*o*-COSAN] (7.5 mgB/kg) was administrated intravenously to MDA-MB-231 breast cancer xenograft model mice, and boron uptake in the target organs was evaluated at 1 and 4 h post-injection. As shown in Fig. 5, significant accumulation of boron was observed in the liver and lungs, while less in the kidney and spleen at 1 h post-injection. After 4 h, a considerable clearance of boron was observed in the target organs, but the tumor boron uptake was high and maintained after 4 h.

We have previously reported a limitation associated with Na[*o*-COSAN], namely the formation of aggregates that predominantly accumulate in the lungs [24]. This warrants detailed investigation to assess the impact of these aggregates on lung function and their potential adverse effects in animals. In the meantime, an alternative approach could be the topical administration of the compound directly to the breast tumor. This would minimize systemic exposure to Na[*o*-COSAN], particularly in the lungs. Additionally, combining Na[*o*-COSAN] with electroporation—similar to the method used by Olaiz et al. [42] with the GB-10 boron compound—could enhance boron uptake in tumors. This would improve T/B and T/N ratios, thereby increasing the effectiveness of BNCT in tumor control.

### Dosimetric studies

#### Calibration curve of XR-RV3 radio-chromic films, using 60Co gamma-rays

**Fig. 6:**
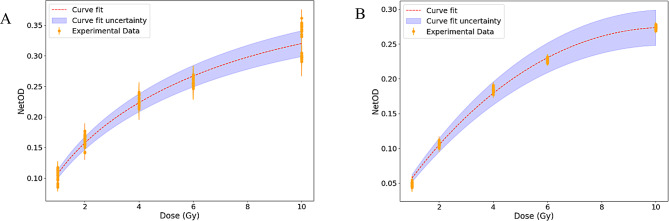
Calibration curve for the ^60^Co source using the unlaminated **(A)** and laminated **(B)** XR-RV3 film

As showed in literature [33, 43], the red channel is optimal for the dose range used (up to 10 Gy). Figure 6 shows the dose-response curve in water for that channel, in the range of 0.5–10 Gy using the ^60^Co source. The values for the parameters $\:A,\:B\:\text{and}\:C$ are presented in Table 1.

**Table 1:** Values of $\:A$ (Gy^− 1^), $\:B$ (Gy^− C^) and $\:C$, and respective uncertainties for the unlaminated and laminated films obtained from Eq. (1)

	Unlaminated film	Laminated film
A (Gy^-1^)	-0.04 ± 0.01	0.065 ± 0.002
B (Gy^-C^)	0.15 ± 0.01	-0.008 ± 0.001
C	0.70 ± 0.04	1.67 ± 0.04

The observed difference between the laminated and unlaminated calibration curves is explained by the presence of color in the polyester and adsive layers. For laminated films the contrast between the background and the irradiated area is lower and the $\:netOD$ will be calculated with lower precision. The similarities in the $\:netOD$ values from different doses are also reflected in the obtained high uncertainties shown in Table 1.

The unlaminated films are also prefered for proton irradiation due to the stopping of the low energy protons in the polyester and adhesive layers. Indeed, the simulations showed a 10^6^ factor reduction in the energy deposited in the active layer for the laminated film when compared with the unlaminated results.

### Dose calculation

The dose in water was scored in the plate well phantom assuming proton intensities of $\:1.5\times\:{10}^{6}$ protons/s and $\:7.42\times\:{10}^{6}$ protons/s, with 10 s and 3 s irradiation times, respectively. The standard deviation of the final dose was obtained by multiplying the standard deviation from the simulation with the square root of the number of particles simulated. The doses in water obtained from the calibration curve and from the simulations are compared in Table 2. The results agree within the uncertainties ranges. In accordance with Sanchez-Parcerisa et al. [32], we conclude that the $\:netOD$ values calibrated with the ^60^Co source can also be used to determine the dose delivered by the proton beam.

**Fig. 7:**
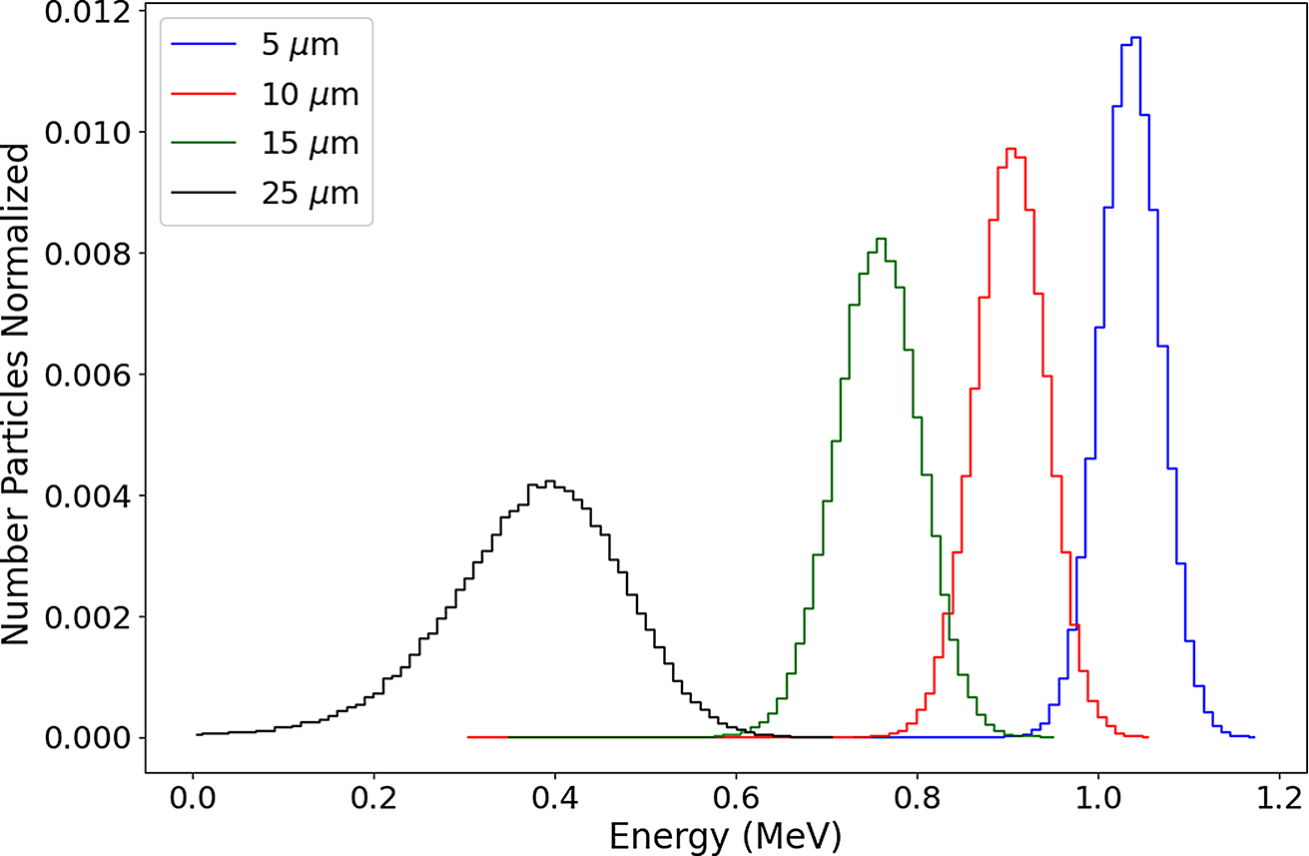
Simulated proton spectra at various dephts on the water cylinder C, defined in Fig. 2

Using the setup in Fig. 1, we simulated the proton energy spectra at various dephts in the water cylinder (C), which are shown in Fig. 7. These results show that the resonance energy is indeed reached inside the irradiated cell layer, between 15 and 25 μm, and are in agreement with previous simpler simulations using the SRIM program (http://www.srim.org/).

**Table 2:** Experimental, netOD and dose (gy) in water, and computational, dose simulated (gy), values with the corresponding uncertainties. The netOD uncertainty was obtained from ImageJ and the dose uncertainty, from the fitted equation, which was obtained based on the IAEA guidelines [44], being approximately 6.62%

Beam intensity (proton/s)	Irradiation time (s)	netOD	Dose measured (Gy)	Dose simulated (Gy)
7.42E + 06	3	0.347 ± 0.003	10 ± 1	8 ± 1
	10	0.465 ± 0.006	25 ± 2	28 ± 2
1.50E + 06	10	0.299 ± 0.002	7 ± 1	6 ± 2

### Radiobiological studies

The efficiency of [*o*-COSAN]^*-*^ was evaluated after irradiation with 8 Gy of protons in MDA-MB-231 cells. The irradiation with protons was carried out assuming that the proton-boron nuclear reaction resonance energy of 675 keV was reached within the cell monolayer.

### Cell survival

**Fig. 8:**
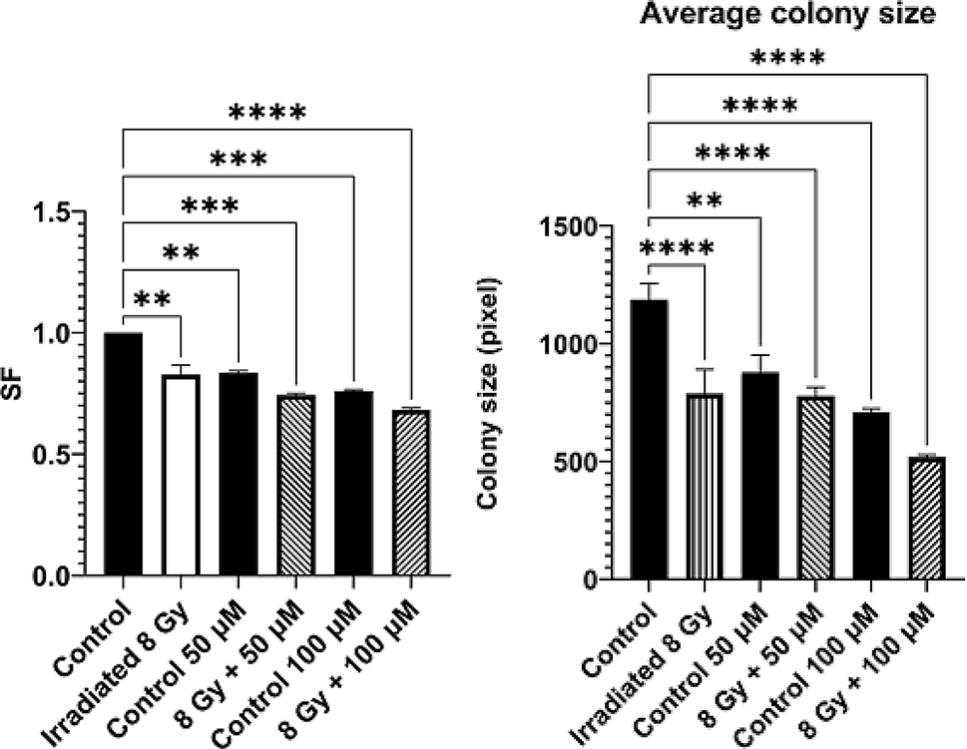
(Left) SF values for the different experimental conditions. Each condition was compared to the control. (Right) Average colony size in pixels of colonies formed in the clonogenic assay. Error bars represent the SEM. ** *p* < 0.01, *** *p* < 0.001, **** *p* < 0.0001. Control showed high significance (****) to all conditions

In terms of cell survival, the results indicate a significant decrease in surviving fraction (SF) after irradiation with 8 Gy, without and with incubation of Na[*o*-COSAN], when compared to the control (non-irradiated cells), Fig. 8 (Left).

Our data highlight the capacity of Na[*o*-COSAN] to increase the radiation effect in MDA-MB-231 cells, because comparing the control irradiated cells with those incubated with Na[*o*-COSAN] and irradiated, a significant decrease is observed (*p* < 0.05), both for 50 µM and 100 µM. Trying to deeply understand that and knowing that alpha-particles induce complex lesions that are more difficult for being repaired, we investigated the area of the colonies formed in each condition. The results are presented in Fig. 8 (Right). Both the presence of [*o*-COSAN]^*-*^ and cellular irradiation significantly impact the average colony size within the samples. Notably, the smallest average colony size was observed in irradiated cells treated with 100 µM Na[*o*-COSAN], exhibiting a 30% decrease compared to their non-irradiated counterparts (*p* < 0.01) and a 40% decrease compared to cells irradiated with protons in the absence of [*o*-Na[COSAN] (*p* < 0.001). All in all, these observations underscore the complex lesions that could be induced by PBFR in the presence of [*o*-COSAN]^*-*^, mainly at 100 µM.

#### Early radiation-induced effects

**Fig. 9:**
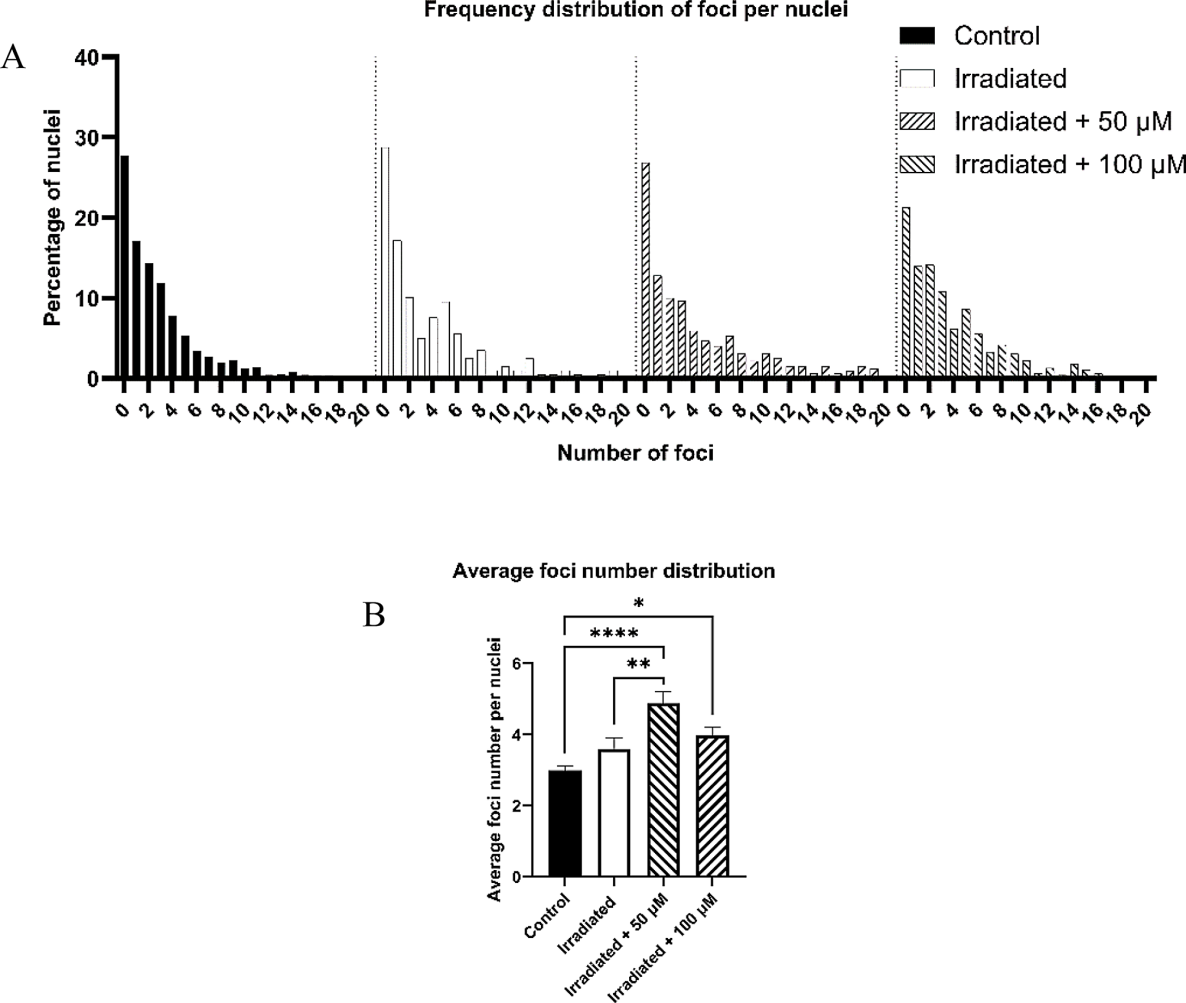
Distribution of γ-H2AX foci across samples. (A) Relative frequency of nuclei with a certain number of foci. (B) Average number of foci. * *p* < 0.05, ** *p* < 0.01, **** *p* < 0.0001

The results on the short-term effects of irradiation in the presence of [*o*-COSAN]^*-*^ are present in Fig. 9. All non-irradiated samples were grouped together as control since similar results were obtained independently of Na[*o*-COSAN] concentration.

From Fig. 9A, we can see in detail how the relative frequency of foci occurred in the different conditions. At first glance, we observe that the percentage of nuclei without foci decreases as the concentration of Na[o-COSAN] increases in irradiated nuclei. Furthermore, there is a greater representation of nuclei with a higher number of foci in these samples when compared to the controls, indicating a stronger short-term radiobiological effect in samples irradiated with Na[*o*-COSAN]. Figure 9B indicates that on average, we observe more foci in irradiated samples, and the presence of [*o*-COSAN]^-^ further significantly increases the number of foci (55% increase in irradiated cells incubated with 50 µM and 27% for cells incubated with 100 µM of Na[o-COSAN]). These results translate into a lower cellular viability as previously discussed, which means that DNA damage imposed colonies smaller that their non-irradiated counterparts, so it can be theorised that these damages took longer times to be repaired or made the cells undergo other less efficient mechanisms in order to survive.

## Discussion

Several types of radiosensitizers have been developed. As chemotherapy and radiotherapy are sometimes used together, some anticancer drugs like cisplatin or paclitaxel have been proposed as radiosensitizers, through several mechanisms. However, high atomic element materials/nanomaterials have been proposed as radiosensitizers due to their high X-ray absorption and unique photoelectric decay process. Although their clinical translation still faces many challenges, in particular nanoparticles, mainly taking into consideration its biodistribution profile, i.e., high uptake in target organs and low uptake in tumors. By its turn, [*o*-COSAN]^-^ with its promising biological profile and biodistribution, i.e., fast clearance in main organs and retention in tumor show to be promising as radiosensitizer to enhance the effect of proton radiation.

Biodistribution studies using an MDA-MB-231 breast cancer xenograft mouse model revealed significant potential for enhancing radiation therapy, because the boron uptake in the tumor was notably high, with a very slow rate of decrease, which is an important result for therapeutic effectiveness. With these findings, we investigate further and explored the potential of the proton boron fusion reaction (PBFR) at the cellular level.

The MDA-MB-231 cells efficiently take up [*o-*COSAN]^-^ even at low concentrations, approximately half of the IC_50_ (137 µM, 24 h), with a predominant nuclear localization of Co. Nuez-Martínez et al. [40] also found higher nuclear accumulation in U87 glioblastoma cells treated with Na[*o*-FESAN], a related Fe metallacarborane This may result in a significantly high ^11^B content (80% of natural boron) in the cell nucleus, which is crucial for enhancing the efficiency of proton irradiation by favoring nuclear proton-boron reactions and consequently causing significant cytogenetic effects. The anionic cobaltabis(dicarbollide) [*o-*COSAN]^-^ cluster is a compact three-dimensional molecule with dimensions of 1.1 nm by 0.6 nm, containing two boron clusters per cobalt atom and has been shown to incorporate into double-stranded DNA (ds-DNA) [24]. These properties suggest that [*o-*COSAN]^-^, which electrostatic intercalates with DNA base pairs [24], is consistent with the high concentration of Co in the nucleus of MDA-MB-231 cells treated with Na[*o-*COSAN]. This intercalation, combined with the high boron content of the [*o-*COSAN]^-^ cluster, could effectively promote proton-boron nuclear reactions within the nucleus by positioning ^11^B atoms close to the genetic material.

The promising results observed for [*o*-COSAN]^-^ uptake in single cells make this compound a strong candidate for further investigation into its induced biological effects. We observed a significant increase in proton-induced cytogenetic effects, both in terms of cell death, assessed as loss of proliferative potential by the clonogenic assay, and in DNA damage induction. The latter was evaluated through the expression of γ-H2AX, a biomarker for DNA double-strand breaks. The markedly higher frequency of γ-H2AX foci levels found with irradiated Na[*o*-COSAN] treated cells compared to proton-irradiated cells indicated that the alpha-particles generated in the nuclear fusion reaction could be responsible for the enhanced biological effect. In addition, the fact that the presence of Na[*o*-COSAN] does not induce new γ-H2AX foci, further corroborates that this increase in DNA lesions is caused by alpha-particles produced from PBFR. Further research shall include a time-dependent analysis of the evolution of the number of γ-H2AX foci as to assess the degree of complexity caused by the produced alpha-particles. In terms of long-term radiobiological effects, our data show that incubation with Na[o-COSAN] leads to minor cytostatic effects, as the SF was lower in treated non-irradiated cells compared with cells under control conditions. Also, proton irradiation in the presence of NA[o-COSAN] further decreased the SF, as it was statistically lower in treated and irradiated cells compared to ther non-irradiated counterparts, which highlights the added effects caused by the PBFR and the produced alpha-particles. This is supported by the decrease in the area of the colonies formed after proton irradiation in the presence of Na[o-COSAN], which was significantly lower in irradiated cells treated with 100 µM of Na[o-COSAN]. The smaller colonies formed by the treated and irradiated cells could be a consequence of the complex DNA lesions caused by alpha particles, so it can be theorised that this damage took longer to repair or that the cells used less effective mechanisms for proliferation and survival. Finally, it should be noted that these radiobiological effects were present in spite of only around 40% of cells being irradiated, which suggests that, in our analysis, non-irradiated cells may lead to an underestimation of the actual damages to the cells. Therefore, future work should focus on how to only assess irradiated cells as a means to overcome this limitation to fully understand the radiosensitizing potential of [o-COSAN]^-^.

The biological effects of radiation are directly related to the absorbed dose making precise dose calculation essential for accurate experimental outcomes when irradiating cultured cells. Known for their ease of use and reliability, radiochromic films are extensively used for dosimetry in both clinical and scientific contexts. We used both laminated and unlaminated XR-RV3 radiochromic films to generate dose calibration curves from ^60^Co irradiation. The dose curves for laminated and unlaminated films provided similar accuracy in dose measurements. The use of laminated films is particularly important when working with research accelerators that produce low energy proton beams. These low energy protons can be stopped by the polymer coating of the film before they penetrate to the active layer, leading to potentially unreliable measurements. Sanchez-Parcerisa et al. [32] have shown that different types of radiochromic films, both laminated and unlaminated, are suitable for dosimetry with both photon and proton beams. Our study also shows that unlaminated XR-RV3 films calibrated with ^60^Co radiation are reliable for routine dosimetry with protons in cell experiments. The dose values for protons, obtained with the ^60^Co dose calibration curve fit, seemed to reach a maximum with the highest fluence value used. This could be due to the red channel values which were used. This channel is optimal up to the 10 Gy range. By contrast, above 40 Gy the green channel shows better results [43]. With the setup implemented in the simulations, we were able to reproduce the dose values determined for the cell irradiation layer within the uncertainties. This validation will allow future simulations, at sub-cellular scales, based on realistic irradiation conditions. In particular, the proton energy spectra was parameterized and we found that the PBFR reaction resonance energy of 675 keV is reached within the target cell layer during irradiation. However, it should be noted that below 1 MeV, the cross-section for this reaction is not currently implemented in the TOPAS (and Geant 4) physics lists. In reality, there is not much experimental data for the ^11^B(p,α)^12^C*→3α reaction at low energies. However, based on recent data [45], we are developing extensions to TOPAS to overcome this limitation [46].

## Conclusions

The high Co content in the nucleus may then reflect high ^11^B content (corresponding to 14.4 ^11^B atoms in 18 total boron atoms per [*o*-COSAN]^−^ cluster). In turn the high ^11^B concentration in the nucleus may amplify the proton irradiation damage due to the proton-boron nuclear reaction, by targeting the DNA more effectively. This is consistent with the cytogenetic effects observed in MDA-MB-231 cells treated with Na[*o*-COSAN], after proton irradiation, characterized by increased DNA damage and reduced cell survival when compared with irradiated untreated cells. Our results emphasize the potential of radiosensitizers to enhance the DMF in proton therapy. Although a beneficial effect of Na[*o*-COSAN] has been confirmed, it was not possible to estimate which process or processes were responsible for the observed experimental effects. One of the most important characteristics of the ionizing radiation (IR), at the micro/nanoscopic level, is the spatial, temporal and spectral aspects of the stochastic nature of the energy deposition processes. The pattern of energy deposition in biological tissues is crucial for understanding radiation-induced biological effects, primarily because it results in the formation of complex lesions that cause clustered DNA damage. The biological importance of clustered DNA damage, a unique signature of radiation-induced DNA damage, has been demonstrated by studies that showed that the repair of lesions within a clustered DNA damage site is compromised when compared with the more common isolated DNA lesions such as single strand breaks and base lesions [47, 48]. Thus, the clustering of physical events (e.g. ionizations and excitations) occurring nearby the radiosensitizer at the nanometre scale may be the most important physical quantities for biologically relevant radiation damage. This is part of our ongoing and future research, which aims to integrate imaging of subcellular distributions into physico-chemical modeling to ultimately achieve precise modeling of biological effects at the level of individual cells. In particular, for PBFR the correct cellular ^11^B placement intensifies the DNA damage, making the nucleus particularly susceptible. Among the possibilities, for the observed enhanced biological effects, are the formation of ROS, α-particles, and hot spots, resulting from the electrons produced near the Na[*o*-COSAN].The hypothesis that the increased cell mortality observed in this study was caused by the produced alpha particles cannot be fully confirmed. However, it remains a possibility, as Monte Carlo simulations indicated that the resonance energy was indeed achieved within the cellular monolayer.

## Data Availability

All the data is reported on the manuscript.

## Abbreviations

DSB: Double-Strand Breaks
LET: Linear Energy Transfer
MC: Monte Carlo
PBFR: Proton Boron Fusion Reaction
PT: Proton Therapy
RBE: Relative Biological Effectiveness
RT: Radiation Therapy
ROS: Reactive Oxygen Species

## References

[1] Siegel et al. 2023 Cancer statistics, CA Cancer. J Clin 73(1) 17–48. 10.3322/caac.21763 36633525

[2] Hanahan D. and Weinberg Hallmarks of cancer: the next generation Cell. 2011; 144(5) 646–674. 10.1016/j.cell.2011.02.013 21376230

[3] Chen et al. Radiotherapy modulates tumor cell fate decisions: a review. Radiat Oncol. 2022;17:196. 10.1186/s13014-022-02171-7 PMC9714175 36457125

[4] Nishimura. Rationale for chemoradiotherapy. Int J Clin Oncol. 2004;9(6):414–20. 15616871 10.1007/s10147-004-0443-z

[5] Sia, et al. Molecular mechanisms of Radiation-Induced Cancer Cell death: a primer. Front Cell Dev Biol. 2020;13 8:41. 10.3389/fcell.2020.00041 PMC7031160 32117972

[6] Jiao et al. Radiation-induced cell death and its mechanisms. Health Phys 123(5) 2022; 376–86. 10.1097/HP.0000000000001601 PMC9512240 36069830

[7] Wang, et al. Cancer Radiosensitizers Trends Pharmacol Sci. 2018;39(1):24–48. 29224916 10.1016/j.tips.2017.11.003

[8] Gong et al. application of Radiosensitizers in Cancer Radiotherapy. Int J Nanomed 16 .2021;1083–102. 10.2147/IJN.S290438 PMC7886779 33603370

[9] Liu. and Chang Proton therapy in clinical practice. Chin J Cancer 30(5). 2011; 315–26. 10.5732/cjc.010.10529 PMC4013396 21527064

[10] Dinesh. Proton therapy for cancer treatment. J Oncol Pharm Pract. 2011;17(3):186–90. 20634263 10.1177/1078155210375858

[11] Paganetti. Proton relative biological effectiveness - uncertainties and opportunities. Int J Part Ther. 2018;5(1):2–14. 30370315 10.14338/IJPT-18-00011.1 PMC6200410

[12] Tian, et al. The evolution of proton beam therapy: current and future status. Mol Clin Oncol. 2018;8(1):15–21. 29399346 10.3892/mco.2017.1499 PMC5772792

[13] Mohan. A review of proton therapy – current status and future directions. Precis Radiat Oncol. 2022;6(2):164–76. 36160180 10.1002/pro6.1149 PMC9499036

[14] Yoon D, et al. Application of proton boron fusion reaction to radiation therapy: a Monte Carlo simulation study. Appl Phys Lett. 2014;105:223507.

[15] Jung et al. 2017 Comparison between proton boron fusion therapy (PBFT) and boron neutron capture therapy (BNCT): a monte carlo study *Oncotarget* 8(24) 39774–39781. 10.18632/oncotarget.15700 PMC5503652 28427153

[16] Khaledi, et al. Is the proton–boron fusion therapy effective? I Radiother Pract. 2021;20(2):153–7.

[17] Cirrone, et al. First experimental proof of Proton Boron Capture Therapy (PBCT) to enhance protontherapy effectiveness. Sci Rep. 2018;8:1141. 29348437 10.1038/s41598-018-19258-5 PMC5773549

[18] Blaha et al. 2021 The Proton-Boron reaction increases the Radiobiological effectiveness of clinical low- and high-energy Proton beams: Novel Experimental evidence and perspectives Front Oncol. 28(11) 682647. 10.3389/fonc.2021.682647 10.3389/fonc.2021.682647 PMC8274279 34262867

[19] Lebedev etal, et al. Radiosensitizing effect of boron enhances the effectiveness of proton therapy in vitro. RAD Conf Proc. 2020;4:60–5.

[20] Manandhar, et al. Effect of boron compounds on the biological effectiveness of proton therapy. Med Phys. 2022;49(9):6098–109. 35754208 10.1002/mp.15824

[21] Ricciardi, et al. A new low-energy proton irradiation facility to unveil the mechanistic basis of the proton-boron capture therapy approach. Appl Sci. 2021;11:11986. 10.3390/app112411986.

[22] Tran et al. 2023 Current State and Prospectives for Proton Boron Capture Therapy *Biomedicines* 11(6) 1727. 10.3390/biomedicines11061727 PMC10296516 37371822

[23] Nuez-Martínez, et al. Boron clusters (ferrabisdicarbollides) shaping the future as radiosensitizers for multimodal (chemo/radio/PBFR) therapy of glioblastoma. J Mater Chem B. 2022;10(47):9794–815. 36373493 10.1039/d2tb01818g

[24] Fuentes et al. 2018 Metallacarboranes on the Road to Anticancer Therapies: Cellular Uptake, DNA Interaction, and Biological Evaluation of Cobaltabisdicarbollide [COSAN] *Chemistry* 22 24(65):17239–17254. 10.1002/chem.201803178 30222214

[25] Ribeiro et al. New Cu(II) complexes with pyrazolyl derived Schiff base ligands: synthesis and biological evaluation. J Inorg Biochem 174. 2017; 63–75. 10.1016/j.jinorgbio.2017.05.011 28623731

[26] Pinheiro P, et al. Cellular targets of cytotoxic copper phenanthroline complexes: a multimodal imaging quantitative approach in single PC3 cells. Metallomics. 2024;16(mfae051). 10.1093/mtomcs/mfae051. 10.1093/mtomcs/mfae051 39510960

[27] Buades, et al. The Mössbauer effect using 57Fe-ferrabisdicarbollide ([o-57FESAN]-): a glance into the potential of a low-dose approach for glioblastoma radiotherapy Inorg. Chem Front. 2022;9:1490–503.

[28] Setilo, et al. Dosimetric comparison between XR-RV3 and EBT2 radiochromic film in megavoltage photon beams. Int J Radiation Res. 2016;14(2):149–52.

[29] Niroomand-Rad, et al. Report 235 - report of AAPM Task Group 235 - Radiochromic Film Dosimetry: an update to TG-55. Med Phys. 2020;47(12):5986–6025. 32990328 10.1002/mp.14497

[30] Belchior, et al. Dose mapping of a ^60^Co irradiation facility using PENELOPE and MCNPX and its validation by chemical dosimetry. Appl Radiat Isot. 2007;66(4):435–40. 18222694 10.1016/j.apradiso.2007.11.017

[31] Reinhardt, et al. Comparison of Gafchromic EBT2 and EBT3 films for clinical photon and proton beams. Med Phys. 2012;39(8):5257–62. 22894450 10.1118/1.4737890

[32] Sanchez-Parcerisa, et al. Radiochromic film dosimetry for protons up to 10 MeV with EBT2, EBT3 and unlaminated EBT3 films. Phys Med Biol. 2021;66:115006. 10.1088/1361-6560/abfc8d 33910190

[33] McCabe, et al. Calibration of GafChromic XR-RV3 radiochromic film for skin dose measurement using standardized X-ray spectra and a commercial flatbed scanner. Am Ass Phys Med. 2012;38(4):1919–30. 10.1118/1.3560422 PMC3078021 21626925

[34] Perl, et al. TOPAS: an innovative proton Monte Carlo platform for research and clinical applications. Med Phys. 2012;39(11):6818–37. 23127075 10.1118/1.4758060 PMC3493036

[35] Pinheiro et al. metallacarboranes for proton therapy using research accelerators: a pilot study. 2023; EPJ Techn Instrum 10 5.

[36] Qi Tan, et al. Dosimetric uncertainties impact on cell survival curve with low energy proton. Physica Med. 2020;76:277–84. 10.1016/j.ejmp.2020.07.005 32738775

[37] Carpenter et al. 2006 CellProfiler: image analysis software for identifying and quantifying cell phenotypes. Genome Biol 7(10) R100. 10.1186/gb-2006-7-10-r100 PMC1794559 17076895

[38] Plesek. Potential applications of the Boron Cluster compounds. Chem Rev. 1992;92:269–78.

[39] Nuez-Martinez, et al. editors. 2021 (a) Cobaltabis(dicarbollide) ([*o*-COSAN]–) as Multifunctional Chemotherapeutics: A Prospective Application in Boron Neutron Capture Therapy (BNCT) for Glioblastoma *Cancers* 13(24) 6367. 10.3390/cancers13246367 PMC8699431 34944987

[40] Nuez-Martínez et al. 2021 (b) Synchrotron-Based Fourier-Transform Infrared Micro-Spectroscopy (SR-FTIRM) Fingerprint of the Small Anionic Molecule Cobaltabis(dicarbollide) Uptake in Glioma Stem Cells. *International Journal of Molecular Sciences* 22(18) 9937. 10.3390/ijms22189937 PMC8466526 34576098

[41] Malaspina DC, et al. How a few help all: cooperative crossing of lipid membranes by COSAN anions phys. Chem Chem Phys. 2023;25:27942. 10.1039/d3cp03614f. 10.1039/d3cp03614f 37823330

[42] Olaiz et al. 2023 Enhancement in the Therapeutic Efficacy of In Vivo BNCT Mediated by GB-10 with Electroporation in a Model of Oral Cancer *Cells 12* 1241. 10.3390/cells12091241 PMC10177359 37174642

[43] Battaglia et al. 2016 Dosimetric response of radiochromic films to protons of low energies in the Bragg peak region. Phys Rev Accelerators Beams 19(6).

[44] FAO/IAEA 2022 Dosimetry for SIT. Standard Operating Procedures for Gafchromic™ Film Dosimetry System for Low Energy X Radiation v. 1.0, Andrew Parker, Kishor Mehta and Yeudiel Gómez-Simuta, editors, Food and Agriculture Organization of the United Nations/International Atomic Energy Agency. Vienna, Austria. 42 pp. https://www.iaea.org/sites/default/files/x-ray-sop-en-excelembedded.pdf

[45] Sikora, et al. A new evaluation of the 11(p,α) αα reaction rates. J Fusion Energ. 2016;35:538–43.

[46] Cirrone et al. Study of the discrepancy between analytical calculations and the observed biological effectiviness in proton boron capture therapy (PBCT). Rad. Applic. 2018; 3: 147–151.

[47] Pastwa P, et al. Repair of radiation-induced DNA double-strand breaks is dependent upon radiation quality and the structural complexity of double-strand breaks. Radit Res. 2003;159:251–61. 10.1667/0033-7587(2003)159[0251:roridd]2.0.co;2 12537531

[48] Wilkinson, et al. The Cellular response to complex DNA damage Induced by Ionising Radiation. Int J Mol Sci. 2023;24:4920. 10.3390/ijms24054920. 36902352 10.3390/ijms24054920 PMC10003081

